# Breakthrough Advances in Beta-Lactamase Inhibitors: New Synthesized Compounds and Mechanisms of Action Against Drug-Resistant Bacteria

**DOI:** 10.3390/ph18020206

**Published:** 2025-02-03

**Authors:** Ya-Si Huang, Hong Zhou

**Affiliations:** 1 Key Laboratory of Basic Pharmacology of Ministry of Education, Zunyi Medical University, Zunyi 563006, China; uiuivtee@163.com; 2 Joint International Research Laboratory of Ethnomedicine of Ministry of Education, Zunyi Medical University, Zunyi 563006, China

**Keywords:** antibiotic resistance, beta-lactamase inhibitors, metallo-beta-lactamases, serine beta-lactamases

## Abstract

Beta-lactam drugs hold a central place in the antibacterial arsenal, and the production of beta-lactamases by drug-resistant bacteria has severely compromised the effectiveness of nearly all available beta-lactams. Therefore, in the face of the increasing threat of drug resistance, the combined use of beta-lactamase inhibitors (BLIs) with beta-lactam antibiotics is crucial for treating infections caused by drug-resistant bacteria. Hence, the development of BLIs has always been a hot topic in the field of medicinal chemistry. In recent years, significant progress has been made in screening active drugs by enhancing the affinity of inhibitors for enzymes and the stability of their complexes, based on the design concept of competitive inhibitors. Here, we review the effects and mechanisms of newly synthesized beta-lactamase inhibitors on various BLIs in recent years, to provide ideas for the development of subsequent beta-lactamase inhibitors.

## 1. Introduction

The World Health Organization (WHO) has listed antibiotic resistance as one of the most challenging public health threats of the 21st century [1]. Bacterial infections are very common in clinical practice, and beta-lactam antibiotics are the most commonly used therapeutic drugs; they play an important role in the treatment of a variety of clinical pathogenic infections [2]. However, under the selective pressure of antibiotics, the phenomenon of bacterial resistance is serious, and its resistance mechanism is mainly related to the production of beta-lactamases encoded by chromosomes or plasmids and the acquisition of PBP2a [3]. At present, the clinical practice often employs a combination of beta-lactamase inhibitors with beta-lactam antibiotics to control infections caused by drug-resistant bacteria [4]. Although most beta-lactamase inhibitors (BLIs) alone are ineffective against bacteria, research has shown that combining inhibitors with antibiotics is a safe and effective treatment, such as amoxicillin–clavulanate, ticarcillin–clavulanate, piperacillin–tazobactam, etc. [5]. With the continuous increase in antibiotic resistance, the effectiveness of traditional beta-lactam antibiotics in treating a variety of clinical infections has been severely affected. To address this challenge, researchers are actively developing new beta-lactamase inhibitors (BLIs) to enhance the potency of existing antibiotics and overcome bacterial resistance.

## 2. Beta-Lactamase

Beta-lactamases (BLAs) are enzymes produced by drug-resistant bacteria that hydrolyze the activity of beta-lactam antibiotics such as penicillins and cephalosporins [6]. Currently, there are more than 2000 known types of beta-lactamases [7], with molecular weights typically ranging from 20 kDa to 50 kDa [8]. According to Ambler’s molecular structure classification, they can be divided into four classes: class A, B, C, and D, based on their amino acid sequences. Class A, C, and D beta-lactamases are called serine-beta-lactamases (SBLs) because they have serine residues in their active sites for catalysis [9]. Class B beta-lactamases are also known as metallo-beta-lactamases (MBLs) because their active sites contain metal ions, especially zinc ions (Zn^2+^) [10]. The activity and catalytic mechanism of these enzymes are closely related to the presence of these metal ions.

### 2.1. MBLs (Class B Beta-Lactamase)

MBLs are of great importance in antibiotic resistance because they are one of the main reasons for resistance to almost all beta-lactam antibiotics. Since the 1990s, with the spread of mobile genetic elements carrying MBL genes among important Gram-negative pathogens, the clinical significance of these enzymes has been increasingly recognized. MBLs are primarily found in pathogens such as *Enterobacteriaceae*, *Pseudomonas aeruginosa*, and *Acinetobacter baumannii*. MBLs can hydrolyze almost all classes of beta-lactam antibiotics, including penicillins, cephalosporins, and carbapenems, the latter of which were once considered the “antibiotics of last resort”. Epidemiological data on MBLs show that they are spreading globally, with a higher prevalence particularly in Asian countries, although the distribution varies by country and region, influenced by local antibiotic usage and hospital practices. The spread of MBLs poses a significant challenge to public health, revealing the inadequate preparedness of global public health systems to address such emergencies [11,12].

Based on molecular structure and sequence homology, metal beta-lactamases (MBLs) are further divided into three subclasses: B1, B2, and B3 [13]. This classification is based on differences in the amino acid sequences of the active sites, zinc ligands, zinc content, ring structures, and substrate profiles. Important MBLs, including IMP (imipenem)-, NDM-, and VIM-types, all belong to the B1 subclass [14,15]. Known B2 class MBLs are relatively few, but some known B2 class MBLs include the following: CphA, which is one of the earliest-discovered B2 class MBLs and originates from *Aeromonas hydrophila*; ImiS, this enzyme is found in *Acinetobacter* species and is capable of hydrolyzing carbapenems [16]. B3 class MBLs are a smaller subclass of MBLs, mainly including L1-type and SMB-1-type. B3 class MBLs are usually found in environmental microorganisms, not in clinically relevant pathogens [17].

NDM enzymes, which are MBLs capable of hydrolyzing almost all beta-lactam antibiotics, are currently one of the most widely distributed and concerning MBLs globally [18]. Researchers recently isolated a *Klebsiella pneumoniae* (*K. pneumoniae*) strain carrying the NDM enzyme from a Swedish patient who had previously received treatment in New Delhi, India. By sequencing the DNA of this *K. pneumoniae*, researchers discovered a new gene for MBLs, later named bla NDM-1, which can hydrolyze almost all beta-lactam antibiotics, including carbapenems that were then considered the last line of defense. NDM enzymes are carried by movable genetic elements such as plasmids and can be transferred between different bacteria, leading to rapid dissemination of drug resistance. Subsequently, subtypes such as NDM-2, NDM-5, and NDM-7 were successively detected [19,20]. Compared with NDM-1, these subtypes of NDM have differences at several key amino acid positions, but their functions are similar. Among them, NDM-5 has even stronger hydrolytic capability for certain β-lactam antibiotics [21]. Structurally, scientists have determined the crystal structure of NDM-1, whose active site contains two zinc ions (Zn^2+^), which are crucial for its catalytic mechanism. The zinc ions are coordinated by the side chain carboxyl oxygen atoms of surrounding amino acid residues, such as histidine (His), glutamic acid (Glu), or aspartic acid (Asp), activating water molecules to attack the beta-lactam ring. In addition to the zinc ions, NDM-1 also includes one or more substrate-binding pockets, which are composed of specific amino acid residues and can accommodate different substrate molecules. The NDM-1 structure may contain flexible loops or loops, and these regions may undergo conformational changes during substrate binding and catalysis [22].

IMP enzymes, also known as Korean metallo-beta-lactamases (Korean MBLs), are among the earliest identified and studied members of the MBLs family; they are also one of the more common MBLs in the Asian region [23]. Their amino acid sequences have unique characteristics compared to other MBLs. Although all MBLs contain two zinc ions as core components of the active site, there may be differences in the exact types and positions of zinc coordinating residues between IMP-type enzymes and other MBLs, such as NDM-type enzymes [24]. The amino acid sequence of IMP enzymes includes some conserved regions (areas where the sequence is highly consistent across different strains) and variable regions (areas with greater sequence diversity). IMP enzymes have specific binding sites within their amino acid sequence, which are crucial for their affinity and catalytic efficiency towards particular substrates. The IMP enzyme family has identified multiple different variants, which differ in their amino acid sequences and sometimes in their geographical distribution, host bacteria, and substrate specificity, including IMP-1, IMP-2, IMP-3, IMP-4, IMP-5, etc. The main differences among these variants lie in their amino acid sequences, especially the residues around the active site, which determine their affinity and hydrolysis efficiency towards different beta-lactam antibiotics. In addition, the prevalence of different IMP variants may vary in different regions of the world, which may be related to their transmission mechanisms, types of host bacteria, and patterns of antibiotic use.

VIM enzymes were initially discovered in Italy but have now been widely distributed globally. VIM typically contains a unique amino acid sequence that distinguishes it from other MBLs. The signature sequence of VIM enzymes may include specific conserved regions, which are similar in all VIM variants (such as VIM-1, VIM-2, VIM-4, VIM-7, VIM-28). The amino acid sequence surrounding its active site may differ from that of NDM enzymes, potentially affecting the enzyme’s affinity and catalytic efficiency towards different substrates. Although both VIM and NDM enzymes rely on two zinc ions for catalysis, there are certain differences in the specific types and positions of zinc-coordinating residues [22].

All MBLs share a common αβ/βα sandwich framework, which forms a central “ββ” sandwich structure between two αβ domains. The active site is located in a shallow groove at the bottom of the ββ sandwich structure, surrounded by several loops. Two loops are particularly important: the L3 loop, which contains hydrophobic residues and is located on one side of the groove, plays a significant role in substrate specificity; the L10 loop, which contains residues Lys171 and Asn180, is involved in substrate binding [10].

B1 and B3 MBLs contain two zinc ions (Zn1 and Zn2) in their active sites and have a relatively shallow groove. These enzymes can hydrolyze all currently available beta-lactam antibiotics, except for monobactams (e.g., aztreonam). The process of hydrolysis of beta-lactam antibiotics by MBLs involves the following steps: (1) Substrate binding and specificity: The first step is controlled by loops L3 and L10, which contain key amino acid residues that interact with the substrate, such as Lys171 and Asn180. (2) Dual Zn(II) ion mechanism: The active site of MBLs contains two Zn(II) ions, located at the M1 and M2 sites [25]. These ions work synergistically, with one Zn1 ion coordinating with a water molecule or hydroxide ion, acting as a nucleophile to attack the carbonyl carbon of the beta-lactam ring, forming a tetrahedral intermediate. This intermediate is stabilized by the Zn(II) ion and carries a negative charge. (3) Proton transfer and cleavage: Another Zn(II) ion or other amino acid residues in the active site participate in proton transfer, promoting the breakdown of the intermediate and the cleavage of the C–N bond. (4) Product release and enzyme readiness: After the C–N bond is broken, the beta-lactam ring opens, and the products formed are released. The enzyme returns to its original state, ready for the next catalytic cycle. In this process, His116, His118, Asp120, His196, Cys221, and His263 may be involved in the coordination process with Zn(II) ions [10].

In contrast, B2 subclass MBLs contain only one zinc ion in their active sites, which are surrounded by a helix, making them less accessible. B2 subclass MBLs, such as CphA from *Aeromonas* spp., have a narrower substrate range and are specifically targeted at carbapenem antibiotics. Subclass B2 MBLs only require a single Zn(II) ion to be fully active. In this mechanism, the Zn(II) ion may facilitate the cleavage of the C–N bond by bridging the nitrogen atom.

### 2.2. Serine-Beta-Lactamases

Serine beta-lactamases are a class of enzymes capable of hydrolyzing beta-lactam antibiotics, including penicillins, cephalosporins, and carbapenems [26]. These enzymes inactivate antibiotics through their catalytic mechanisms, leading to bacterial resistance to these drugs. Serine beta-lactamases typically consist of two domains: a predominantly α-helical domain and an α/β domain. The active site is located in the cleft between these two domains, often containing a catalytic triad composed of a serine, a basic amino acid (such as lysine), and an acidic amino acid (such as aspartate), which is responsible for the enzyme’s catalytic activity. Based on amino acid sequence homology and catalytic mechanisms, serine beta-lactamases are classified into classes A, C, and D. These classes of enzymes have highly conserved active site residues, despite potentially significant differences in their amino acid sequences. Different classes of serine beta-lactamases have varying hydrolytic capabilities towards different beta-lactam antibiotics. For example, class A enzymes are typically active against penicillins, cephalosporins, and carbapenemase, while class C enzymes also have activity against cephalosporins and monobactams. Extended Spectrum Β-Lactamases (ESBLs) are one of the most important classes of beta-lactamases, primarily produced by Gram-negative bacilli, with *K. pneumoniae* being the most common among the *Enterobacteriaceae* family, accounting for 16.9% to 75.0% of ESBL-producing *Enterobacteriaceae*. Other common bacteria include *Escherichia coli* (*E. coli*), *Klebsiella oxytoca*, *Enterobacter cloacae*, *Salmonella*, and *Enterobacter aerogenes*. According to Ambler classification, ESBLs mainly belong to class A and class D enzymes, with class A enzymes including types such as TEM and SHV, while class D enzymes primarily consist of OXA-type enzymes. These enzymes can hydrolyze penicillins, cephalosporins, and monobactams, thereby rendering these antibiotics inactive against infections. OXA-48 and OXA-23 are two significant class D carbapenemases that hydrolyze carbapenem antibiotics, leading to bacterial resistance. OXA-48 was first discovered in Turkey in 2001 and is primarily found in Enterobacteriaceae, such as Escherichia coli and Klebsiella species, and has since spread globally, particularly in the Middle East and Europe. OXA-48 hydrolyzes the β-lactam ring of carbapenems, rendering them ineffective, and is highly efficient against imipenem and meropenem, posing a serious threat to the treatment of infections caused by Enterobacteriaceae. OXA-23 was first identified in France in 2001 and is mainly found in Acinetobacter baumannii, a significant hospital-acquired pathogen with resistance to multiple antibiotics. OXA-23 also hydrolyzes the β-lactam ring of carbapenems, making *A. baumannii* highly resistant to these antibiotics, thereby increasing treatment challenges and patient mortality. The detection of both enzymes typically relies on molecular biology techniques, such as PCR and gene sequencing, as their phenotypic characteristics are similar to other β-lactamases and cannot be accurately identified through routine antibiotic susceptibility testing. The emergence and spread of OXA-48 and OXA-23 pose significant challenges to clinical treatment, necessitating more effective detection and identification methods, as well as new therapeutic strategies to combat these resistant bacteria.

#### 2.2.1. Class A Beta-Lactamase

Class A beta-lactamases are clinically very important because they are common resistance mechanisms in many Gram-negative and Gram-positive bacteria, especially in *E. coli* and *K. pneumoniae*. Some well-known class A beta-lactamases include KPC, TEM-1 (widely distributed among *Enterobacteriaceae*), SHV (found in various Gram-negative bacilli), and CTX-M (prevalent among *Enterobacteriaceae*). The genes of class A beta-lactamases are often located on movable genetic elements, such as plasmids, which allows them to be transferred between different bacteria, increasing the spread of resistance genes. Class A beta-lactamases hydrolyze the beta-lactam ring through a bi-molecular reaction mechanism involving a covalent intermediate, and they are usually active against penicillins and cephalosporins but have weak or no activity against carbapenems. This process includes acylation (the active serine residue of the enzyme attacks the carbonyl carbon of the beta-lactam ring) and deacylation (a water molecule attacks the covalent intermediate). The active sites of these enzymes typically contain a catalytic triad composed of a serine (Ser), an acidic amino acid (usually aspartate Asp or glutamate Glu), and a basic amino acid (usually lysine Lys) [27]. Currently, clinically used inhibitors of serine beta-lactamases include clavulanic acid, sulbactam, and tazobactam. These inhibitors protect antibiotics from hydrolysis by binding to the enzyme’s active site, thus preventing the enzyme from degrading the antibiotic [28]. Structurally, BLIs each have their own characteristics. For example, clavulanic acid is an oxa-penicillin class beta-lactamase inhibitor that contains a beta-lactam ring in its structure, capable of forming a covalent bond with beta-lactamases, thereby inhibiting their activity. Sulbactam is a penicillin sulfone class inhibitor, structurally similar to clavulanic acid, but with an additional sulfonic acid group, enhancing its ability to inhibit certain beta-lactamases. Tazobactam is a beta-lactamase inhibitor containing a 1,3-oxazole structure, capable of binding to the active site of beta-lactamases to prevent the hydrolysis of antibiotics. Avibactam is a non-beta-lactam class inhibitor with a bicyclic structure, capable of inhibiting a variety of beta-lactamases, including KPC enzymes. Relebactam is a new type of beta-lactamase inhibitor, structurally similar to tazobactam, but with stronger inhibitory activity. Vaborbactam is a cyclic boronate structure beta-lactamase inhibitor, capable of inhibiting class A and C beta-lactamases, including KPC. Taniborbactam is a bicyclic boronate structure beta-lactamase inhibitor, capable of inhibiting a variety of beta-lactamases, including metallo-beta-lactamases. In 2015, avibactam, a good class A beta-lactamase inhibitor, was approved. Currently there are also others approved, such as relebactam or vaborbactam.

#### 2.2.2. Class C Beta-Lactamase

The clinical importance of class C beta-lactamases is increasing as their presence in various bacteria leads to resistance to commonly used antibiotics, especially among *Enterobacteriaceae*, such as *E. coli*, *K. pneumoniae*, *E. aerogenes*, and *S. marcescens*. These enzymes have good hydrolytic activity against cephalosporins and can also hydrolyze some penicillins, but their activity against carbapenems is usually weak. Class C was represented by AmpC (Ampicillin-class C beta-lactamase), P99 (*Enterobacter cloacae* chromosome-encoded beta-lactamase 99), PDC-1 (*Pseudomonas aeruginosa* chromosome-encoded beta-lactamase 1), and CMY (Cephalosporinase CMY). A well-known class C beta-lactamase is CMY-2, an enzyme found in various Gram-negative bacilli, including strains resistant to multiple antibiotics [29]. The genes of class C beta-lactamases can also be located on movable genetic elements, such as plasmids and transposons, which facilitate the spread of resistance genes among bacterial populations. Class C beta-lactamases are usually sensitive to some beta-lactamase inhibitors like clavulanic acid, but their sensitivity to other inhibitors like tazobactam may be lower. The three-dimensional structures of some class C beta-lactamases have been resolved, providing an in-depth understanding of their catalytic mechanisms and substrate specificity.

#### 2.2.3. Class D Beta-Lactamase

Class D beta-lactamases are a relatively special group of serine beta-lactamases that have hydrolytic activity against a wide range of beta-lactam antibiotics, including penicillins, cephalosporins, and carbapenems [30]. The genes of class D beta-lactamases can also be located on movable genetic elements, which promotes the spread of resistance genes among bacterial populations. OXA (Oxacillinase) type β-lactamases, which are typical representatives of class D beta-lactamases, include those with a reduced spectrum, those exhibiting an ESBLs phenotype, and carbapenemase OXAs, and they are found in various bacteria, including *Acinetobacter* and *Enterobacteriaceae* [31]. However, they differ from other classes of beta-lactamases in structure and catalytic mechanism, usually comprising an N-terminal penicillin-binding protein (PBP) domain and a C-terminal beta-lactamase domain. This structure makes their catalytic mechanism different from that of class A and C beta-lactamases. These enzymes catalyze through a covalent intermediate, which involves a unique N-carboxymethylated lysine residue that plays a key role in the enzyme’s active site. This N-carboxymethylated lysine residue acts as a nucleophile to activate water molecules in the acylation reaction and helps water molecules attack the covalent intermediate in the deacylation reaction. Class D beta-lactamases are generally insensitive to traditional beta-lactamase inhibitors (such as clavulanic acid, tazobactam, and sulbactam), making them more difficult to inhibit, thus posing a challenge to treatment.

## 3. Beta-Lactamase Inhibitors

**Figure 1:**
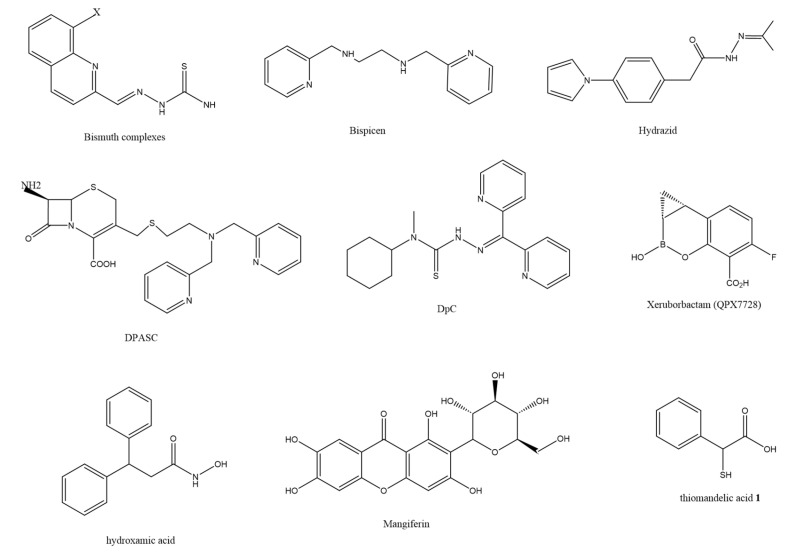
The structures of representative β-lactamase inhibitors.

The clinical deployment of β-lactamase inhibitors is a pivotal development in tackling multidrug-resistant Gram-negative pathogens. Notably, the synergistic use of inhibitors such as avibactam, relebactam, and vaborbactam with beta-lactam antibiotics introduces innovative strategies against infections that are resistant to traditional treatments. These inhibitors curtail bacterial beta-lactamase production, thereby safeguarding the antimicrobial potency of beta-lactam antibiotics from enzymatic degradation. The following provides a detailed look at each. Avibactam: As a non-beta-lactam beta-lactamase inhibitor, avibactam is effective against a spectrum of beta-lactamases, including ESBLs and KPC enzymes. Its combination with ceftazidime significantly boosts the drug’s antimicrobial efficacy against multidrug-resistant Enterobacteriaceae. Relebactam: This novel β-lactamase inhibitor has the capability to suppress a wide array of beta-lactamases, encompassing class A and class C enzymes, as well as carbapenemases. When co-administered with imipenem/cilastatin, relebactam potently neutralizes multidrug-resistant bacterial strains. Vaborbactam: A cyclic boronate non-beta-lactam beta-lactamase inhibitor, vaborbactam exhibits particularly robust activity against class A and class C beta-lactamases. Its combination with meropenem strengthens the antibiotic’s impact on KPC-producing Enterobacteriaceae. The strategic co-application of these beta-lactamase inhibitors enhances antibiotic efficacy and retards the emergence of drug resistance, which is vital for managing severe infections from multidrug-resistant Gram-negative bacteria. This approach empowers medical professionals to combat drug-resistant strains more effectively, thereby conserving and prolonging the utility of existing antibiotics—a development of profound importance to global public health. In recent years, based on the design concept of competitive inhibitors, significant progress has been made in the screening of active drugs by enhancing the affinity of inhibitors for enzymes and the stability of their complexes. Here, we review the inhibitors and mechanisms of each class of β-lactamases in recent years, providing ideas for the development of subsequent beta-lactamase inhibitors (the structures of representative β-lactamase inhibitors are shown in Figure 1).

### 3.1. Class B Beta-Lactamase Inhibitors (MBLs Inhibitors)

Traditional BLIs, such as tazobactam and sulbactam, are only effective against many class A enzymes, but they have been proven ineffective against strains expressing multiple ESBLs, serine carbapenemases (KPC), and class B, C, or D beta-lactamases. The development of new beta-lactamase inhibitors will be a primary focus of future research. As class B enzymes include metal ions, usually one or two zinc ions for their activities, they are called MBLs. The development of MBL inhibitors is of great significance. On the one hand, by being co-administered with existing beta-lactam antibiotics, MBL inhibitors can enhance the antibacterial effects of these drugs against resistant strains. On the other hand, they can suppress the activity of metallo-beta-lactamases in bacteria, thereby reducing bacterial resistance to beta-lactam antibiotics. This is crucial for treating infections caused by drug-resistant strains, which are often more complex to treat and have lower success rates. MBL inhibitors can improve therapeutic outcomes and enhance the health prognosis for patients. Traditional beta-lactamase inhibitors, such as clavulanic acid, sulbactam, and tazobactam, are primarily designed to target serine beta-lactamases. They inhibit the enzyme’s activity by forming covalent bonds with the active serine residues of the enzyme. However, these traditional inhibitors are usually ineffective against MBLs, as the active center of MBLs contains one or two zinc ions, not serine residues. Therefore, the development of new inhibitors specifically targeting MBLs has always been a hot topic in research.

As of now, several MBL inhibitors are undergoing clinical research or have entered the late stages of clinical trials, such as VNRX-5133 (Taniborbactam) [32], ME1071[33], and RPX7009 [34,35,36]. There are still many newly synthesized compounds that have shown good in vitro and in vivo inhibitory effects on MBLs.

#### 3.1.1. NDM Inhibitors

Extensive efforts have been made to develop inhibitors of NDM-1. Lots of newly synthesized compounds exhibit inhibitory activity against NDM-1, such as taniborbactam, quinolinyl sulfonamides, thiosemicarbazones, dexrazoxane, embelin, candesartan, cilexetil, nordihydroguaiaretic acid α-lipoic acid, methimazole, withaferin A, mangiferin, bismuth complexes, risedronate, methotrexate, D-captopril, polypyridine ligands, thiosemicarbazones, etc. (Table 1, Table 2 and Table 3). 

Researchers have evaluated the effects of various compounds on NDM-1’s enzymatic activity, finding that certain compounds, such as quinolinyl sulfonamides, can inhibit NDM-1 to a significant degree, with an IC_50_ value of 0.02 μM. Molecular docking studies further suggest these compounds’ direct binding capacity to the NDM-1 enzyme. By analyzing the RMSD and RMSF values of the protein–ligand complex, the structural stability and flexibility post-binding were assessed, revealing a stable, low RMSD, minimal RMSF fluctuations, sustained hydrogen bonding, and negative binding free energy, all of which indicate a stable ligand–NDM-1 complex, corroborating the experimental findings.

In a specific study, 38 novel benzo or pyrido[d][1,2]selenazol-3(2H)-one derivatives were synthesized based on the NDM-1 protein structure and structure–activity relationship insights. Notably, compound 15l showed significant synergistic antibacterial effects when combined with meropenem against NDM-1-positive carbapenem-resistant *Enterobacteriaceae* (CRE) isolates, particularly clinical CRE isolates, with FIC indices ranging from 0.0625 to 0.25. Molecular docking studies indicated a strong binding affinity of compound 15l for NDM-1. The design of these derivatives considered the crystal structure of NDM-1’s catalytic domain and the interaction between Ebselen—a known covalent inhibitor of NDM-1—and NDM-1 itself. Key residues Lys211 and Asn220, potential hydrogen bond donors near NDM-1’s active site, were targeted by introducing hydrogen bond acceptors in the design, such as replacing the phenyl ring with a pyridine ring to enhance these interactions. Additionally, hydrophobic groups were introduced into the second phenyl ring to interact with the hydrophobic patch outside the active site, composed of Met67, Val73, Leu65, and Tyr93. Polar groups, including primary amines and quaternary ammonium groups, known to enhance antibacterial activity, were also incorporated. Molecular docking predicted the binding model of compound 15l with NDM-1, revealing that the N–Se bond of 15l breaks, allowing the Se atom to form a stable Se–S covalent bond with Cys208’s SH group. The compound’s amide group and the pyridine ring’s N atom form hydrogen bonds with Lys211 and Asn220, respectively. ESI-MS analysis confirmed the covalent binding of 15l to NDM-1. Collectively, these 1,2-benzo or pyrido[d][1,2]selenazol-3(2H)-one derivatives inhibit NDM-1 activity through covalent binding, hydrogen bonding, hydrophobic interactions, and polar group introduction, thereby potentiating the antibacterial effects of meropenem against NDM-1-producing strains. Caburet et al.’s study evaluated the inhibitory activity of Aurones derivatives using spectrophotometric enzyme assays and found that two compounds (**27** and **57**) exhibited low *Ki* values (1.7 and 2.5 µM, respectively) and showed strong inhibitory activity against NDM-1.

Xeruborbactam, a novel boron-based β-lactamase inhibitor, is advancing through clinical development for the oral treatment of infectious diseases in tandem with β-lactam antibiotics. Comprehensive research has confirmed its broad-spectrum inhibitory activity against a range of beta-lactamases both in vitro and in vivo, effectively neutralizing serine beta-lactamases from classes A–D and metal beta-lactamases, such as ESBLs, KPCs, and OXAs [37]. Xeruborbactam has been shown to boost the potency of various beta-lactam antibiotics against challenging pathogens like carbapenemase-producing *Enterobacterales* (CPE), carbapenem-resistant *Acinetobacter baumannii* (CRAB), and *P. aeruginosa* [38,39]. Phase I clinical trials have assessed Xeruborbactam, administered as an oral prodrug (QPX7831) in combination with cefepime, for safety, tolerability, and pharmacokinetics in healthy adults. The findings demonstrate that Xeruborbactam’s oral prodrug is safe and well tolerated across all tested dosages, with plasma AUC and Cmax values rising in conjunction with dosage increments. The drug’s exposure, as measured by 24-h free AUC, surpasses the anticipated PK-PD thresholds, endorsing a once-daily administration regimen. Xeruborbactam presents advantageous pharmacokinetic properties, highlighted by an extended elimination half-life and minimal plasma clearance. It is formulated for both intravenous and oral administration, catering to both hospital and ambulatory care settings. Furthermore, studies evaluating Xeruborbactam’s oral prodrug in combination with cefepime among subjects with renal impairment have been conducted to assess safety and pharmacokinetics, reinforcing the case for Xeruborbactam’s continued clinical development [40].

**Table 1:** Evaluation of MIC reduction capabilities of NDM inhibitors.

Compounds	Substrates	Enzymes	Activity (Reduced MIC by)	Pathogens	Ref.
Taniborbactam	Cefepime, ceftazidime, imipenem, tebipenem, and cefiderocol	NDM-1	32, 133, and 33 fold	EC	[41]
α-lipoic acid, methimazole	Meropenem	NDM-1	16 and 4 fold	EC	[42]
Bismuth complexes	Meropenem	NDM-1	128 fold	KP	[43]
Polypyridine ligands	Meropenem	NDM-1	8–128 fold	KP, SM, *Enterobacter cloacae*	[44]
Thiosemicarbazones	Ampicillin, cefazolin, meropenem	NDM-1	4–32, 4–32, and 4–8 fold	EC	[45]
N-acylhydrazones	Meropenem	NDM-1	4–16 fold	EC, KP	[46]
ANT2681	Meropenem	NDM-1, NDM-4, NDM-5, NDM-7	16–512 fold	EC	[47]
Thiosemicarbazones	Meropenem	NDM-1, ImiS (B2)	8–32 fold	EC, PK	[48]
Dipyridyl-substituted thiosemicarbazone	Meropenem	NDM-1, VIM-2, IMP-1, ImiS (B2), Li (B3)	32 fold	EC, PK	[49]

EC: *Escherichia coli*; KP: *Klebsiella pneumoniae*; AB: *Acinetobacter baumannii*; SM: *Stenotrophomonas maltophilia*; MIC: minimum inhibitory concentration; IC_50_: half maximal inhibitory concentration; FICI: fractional inhibitory concentration index.

**Table 2:** Analysis of IC_50_ values of NDM inhibitors.

Compounds	Substrates	Enzymes	Activity IC_50_ (μM)	Pathogens	Ref.
Ag2O2@BP-MT@MM	Meropenem	NDM-1	0.24	KP	[50]
Risedronate, Methotrexate, D-captopril	Nitrocefin, ampicillin, cefotaxime, imipenem, meropenem	NDM-1	24.6, 29.7, 11.8	EC	[51]
Xeruborbactam	Meropenem, cefepime	NDM-1, NDM-9	0.24–1.2	EC, KP	[52]
Hydroxamates	Meropenem, cefazolin	IMP, NDM-1, VIM-2, Li, ImiS (B2)	0.1–0.23	Not specified	[53]

EC: *Escherichia coli*; KP: *Klebsiella pneumoniae*; IC_50_: half maximal inhibitory concentration.

**Table 3:** FICI values of NDM inhibitors.

Compounds	Substrates	Enzymes	Activity (FICI)	Pathogens	Ref.
Thiosemicarbazones	Meropenem	NDM-1	0.34	*E. cloacae*	[54]
Withaferin A, mangiferin	Imipenem	NDM-1	0.0625–0.5	AB	[55]
1,2-Isoselenazol-3(2H)-one derivatives	Meropenem	NDM-1	0.0625–0.25	carbapenem-resistant *Enterobacteriaceae*	[56]

AB: *Acinetobacter baumannii*; FICI: fractional inhibitory concentration index.

#### 3.1.2. IMP and VIM Inhibitors

By targeting MBLs such as VIM and IMP, scientists have been actively researching and developing effective inhibitors. These inhibitors are designed to bind to the enzyme’s active site, thereby preventing the hydrolysis of antibiotics and restoring or enhancing their antimicrobial activity. By examining the three-dimensional structures of VIM and IMP, scientists have engineered small molecule inhibitors that can specifically target the enzyme’s active site. Certain inhibitors have been designed for VIM and IMP, such as Taniborbactam. Taniborbactam is one of the most advanced BLIs in the clinical phase, having proceeded to clinical trial stages to evaluate its safety and efficacy. Taniborbactam, previously designated as VNRX-5133, is an innovative bicyclic boronate BLI with the capacity to neutralize both serine beta-lactamases across Ambler classes A, C, and D, and MBLs within Ambler class B, including NDM and VIM, while sparing IMP. This inhibitor holds promise as a therapeutic adjunct to cefepime, providing a potential treatment avenue for individuals battling severe bacterial infections from hard-to-treat, drug-resistant Gram-negative pathogens. It is particularly beneficial for those infections caused by metalloenzyme-producing strains that are resistant to carbapenems, such as carbapenem-resistant *Enterobacterales* (CRE) and carbapenem-resistant *P. aeruginosa* (CRPA). Laboratory studies have demonstrated that the cefepime–taniborbactam combination is effective against a broad spectrum of CRE and CRPA strains, whether they harbor carbapenemase production or not, and it shows efficacy against strains that are multidrug-resistant (MDR), as well as those resistant to ceftazidime–avibactam, meropenem–vaborbactam, and cefoperazone–sulbactam. The pharmacokinetic profile of taniborbactam is consistent and dose-dependent, adhering to a linear model that remains stable when co-administered with cefepime. The active center of MBLs contains zinc ions, which are essential for the catalytic activity of the enzyme. Therefore, the development of inhibitors that can effectively inhibit MBLs, especially those that can chelate or strip the Zn^2+^ at the active center of MBLs, has become an important means to combat infections caused by drug-resistant bacteria. There are also chemically synthesized compounds that have shown promising inhibitory effects not only against NDM enzymes but also against VIM and IMP MBLs, which probably inhibit the activity of Zn^2+^ (Table 4, Table 5 and Table 6). For example, researchers have developed compounds based on the 1,2,4-triazole-3-thione scaffold, which can bind to the dual-zinc catalytic sites of MBLs in a novel manner. In particular, the compounds coordinate with Zn^2+^ ions through their carboxylate groups, effectively inhibiting the activity of VIM-type enzymes. Similarly, researchers have used the metal-binding pharmacophore (MBP) click chemistry approach to synthesize a series of new broad-spectrum MBL inhibitors. These compounds also exert their inhibitory effect by coordinating with Zn^2+^ ions at the active site of MBLs. The co-crystal structure analysis mentioned in the article further confirms the importance of MBP when binding to MBLs. The compounds all exert their inhibitory effect by targeting the Zn^2+^ domain of MBLs. This mode of action involves the formation of stable coordination bonds between the metal-coordinating groups in the compounds (such as carboxylate groups, phosphonic acid, etc.) and Zn^2+^ ions, thereby disrupting the enzyme’s catalytic mechanism and reducing its hydrolysis of beta-lactam antibiotics.

**Table 4:** Evaluation of MIC reduction capabilities of VIM and IMP inhibitors.

Compounds	Substrates	Enzymes	Activity (Reduced MIC by)	Pathogens	Ref.
ZN148	Meropenem	VIM-2	2 fold	PA, AB	[57]
1,4,7-triazacyclononane-1,4,7 triacetic acid	Meropenem	IMP-1	9–11 fold	EC, KP, *E. cloacae*	[58]
Cephalosporin-Tripodalamine Conjugate	Meropenem	IMP-4	16–512 fold	EC	[35]
Polyimidazole ligands	Meropenem, aztreonam	VIM-1, IMP-1	4 fold	EC, KP	[59]
N-Aryl Mercaptopropionamides	Imipenem	IMP-7, VIM-1	256 fold	EC	[60]
N-Sulfamoylpyrrole-2-carboxylates	Meropenem	VIM-1, VIM-2, IMP-1	170 fold	EC, KP	[61]
Cephalosporin Conjugates	Meropenem	IMP-1, IMP-28, VIM-2	64 fold	EC, KP, *E. cloacae*	[62]
1,2,4-Triazole-3-Thione Analogues	Meropenem	VIM-1, VIM-2, VIM-4, IMP-1	4–16 fold	EC	[63]
1H-imidazole-2-carboxylic acidderivatives	Meropenem	VIM-2, VIM-1, VIM-5, IMP-1	64 fold	EC	[64]

EC: *Escherichia coli*; KP: *Klebsiella pneumoniae*; AB: *Acinetobacter baumannii*; MIC: minimum inhibitory concentration.

**Table 5:** Analysis of IC_50_ values of VIM and IMP inhibitors.

Compounds	Substrates	Enzymes	Activity IC_50_ (μM)	Pathogens	Ref.
Alkylthio-substituted aliphatic thiols 3	imipenem	VIM-1	0.3 and 0.02	EC	[65]
Cephalosporin derivatives	meropenem, doripenem	IMP-1, VIM-27	130	*Acinetobacter*	[66]
4-Alkyl-1,2,4-triazole-3-thione analogues	meropenem	VIM-2, VIM-4	0.27	KP	[67]

EC: *Escherichia coli*; KP: *Klebsiella pneumoniae*; IC_50_: half maximal inhibitory concentration.

**Table 6:** FICI values of VIM and IMP inhibitors.

Compounds	Substrates	Enzymes	Activity (FICI)	Pathogens	Ref.
H2dpa derivatives	meropenem	IMP-1, VIM-2	0.07–0.18	EC	[68]
Two catechol-conjugated compounds	imipenem	VIM-1, IMP-7	0.045–4.3	KP	[69]

EC: *Escherichia coli*; KP: *Klebsiella pneumoniae*; FICI: fractional inhibitory concentration index.

### 3.2. Serine Beta-Lactamases Inhibitor

SBLs are a class of enzymes capable of hydrolyzing beta-lactam antibiotics, including penicillins, cephalosporins, and carbapenems. SBLs exert their catalytic action through serine residues in their active sites, hydrolyzing the beta-lactam ring of antibiotics, which leads to the inactivation of the antibiotics. This hydrolytic action is one of the significant causes of bacterial resistance to beta-lactam antibiotics. The active sites of SBLs contain one or more serine residues that directly participate in the catalytic reaction. The catalytic mechanism of SBLs typically involves the following steps: (1) Nucleophilic attack: The serine residue in the active site acts as a nucleophile, attacking the carbonyl carbon atom of the beta-lactam ring. (2) Acyl enzyme intermediate formation: The nucleophilic attack results in the cleavage of the beta-lactam ring, forming an acyl enzyme intermediate. (3) Hydrolysis: A water molecule attacks the acyl enzyme intermediate, leading to the hydrolysis of the intermediate and the release of the final product from the enzyme.

As bacterial resistance continues to rise, research on SBLs is progressively deepening, with the development of new inhibitors emerging as a central focus of study. Below is an introduction to some compounds and their lead compounds that have been synthesized chemically and possess the ability to inhibit serine beta-lactamases, along with a preliminary exploration of their mechanisms of action (Table 7 and Table 8).

From Arakawa’s study, we can see that the compound 2,5-diethyl-1-methyl-4-sulfamoylpyrrole-3-carboxylic acid (SPC) was synthesized through chemical modification of the previously discovered 2,5-dimethyl-4-sulfamoylfuran-3-carboxylic acid (SFC). Researchers, based on the binding mode of SFC with MBLs, specifically synthesized SPC to enhance its inhibitory effect on a variety of clinically relevant MBLs, including IMP-type, NDM-type, and VIM-type enzymes. In addition, SPC exhibits strong inhibitory effects against enzymes TMB-2, DIM-1, SIM-1, and KHM-1. For *E. coli* producing TMB-2, SPC reduced the MIC of meropenem from 16 μg/mL to 0.031 μg/mL (a 512-fold decrease). For *E. coli* producing DIM-1, SPC reduced the MIC of meropenem from 8 μg/mL to 0.063 μg/mL (a 128-fold decrease). Alsenani et al.’s study investigated a novel series of non-covalent broad-spectrum β-lactamase inhibitors—azetidinimines—and found that compound 7dfm exhibited strong inhibitory activity against the three clinically relevant carbapenemases NDM-1, OXA-48, and KPC-2 at the submicromolar level, with *Ki* values of 0.07 mM, 0.07 mM, and 0.28 mM, respectively, demonstrating highly effective inhibitory effects. Structurally, SPC binds to MBLs through several key structural features to exert its inhibitory effect. (1) Sulfamoyl group: This group is capable of coordinating with two zinc ions (Zn1 and Zn2) in the active site of MBLs. Such coordination is crucial for the inhibitor’s enzyme-inhibiting activity. (2) Carboxylate group: The carboxylate group can form a coordination bond with a zinc ion (usually Zn2) and can also form hydrogen bonds or other types of interactions with positively charged amino acid residues near the active site, such as Lys224 or Arg228. (3) Heterocyclic core structure: The heterocyclic core structure of the compound (such as a pyrrole ring) provides support for the sulfamoyl and carboxylate groups and helps the compound to precisely match the active site of MBLs. (4) Additional carbon atom framework: The additional carbon atom framework of SPC (such as ethyl groups at positions C2 and C5) enhances the hydrophobic interactions with the hydrophobic region surrounding the active site of MBLs, thereby increasing the potency and selectivity of the inhibitor. (5) Compounds like SPC are able to interact with the conserved regions of the active site of MBLs, which means they are not only inhibitory to specific types of MBLs but may also be effective against a variety of different MBLs.

## 4. Conclusions

This review highlights the critical role of β-lactamase inhibitors (BLIs) in fighting antibiotic resistance, a major global health threat. BLIs enhance the efficacy of existing antibiotics and curb the spread of drug-resistant bacteria, offering new treatment strategies (Table 9). Their safety, efficacy, and clinical trial progress were discussed. This review also underscores the importance of understanding antibiotic-β-lactamase interactions through structural analysis for designing more potent inhibitors. Despite advancements, challenges remain, including the need for broad-spectrum inhibitors due to β-lactamase mutability, and the assessment of new antibiotics and BLI combinations in clinical trials. PK/PD properties of BLIs are essential for optimizing treatment, and toxicity and side effects of inhibitors require attention. Overall, BLI research holds promise for overcoming drug resistance. Continued research and innovation, along with the support of governments, industries, and the public, are needed to develop more effective inhibitors and combat the rising issue of drug resistance.

## Figures and Tables

**Table 7:** Evaluation of MIC reduction capabilities of serine beta-lactamases inhibitors.

Compounds	Substrates	Enzymes	Activity (Reduced MIC by)	Pathogens	Ref.
Sulfamoyl Heteroarylcarboxylic Acids	Meropenem	TMB-2, SPM-1, DIM-1, SIM-1, KHM-1	128 fold	EC	[70]
Pep3 peptide	Meropenem	TEM-1	64 fold	EC, *E. cloacae*	[71]
Xeruborbactam	Cefepime, ceftolozane, ceftriaxone, aztreonam, oiperacillin, ertapenem	KPC-2, CTX-M-14, CTX-M-15, SHV-12, TEM-10	8 fold	*Enterobacterales*	[72]
Durlobactam	Sulbactam	TEM-1,KPC-2, ADC-7, OXA-24	32 fold	*Acinetobacter*	[73]
1,5 disubstituted, 1,4,5 trisubstituted triazole DBOs	Aztreonam	KPC-2	32,000 fold	EC	[74]
Aromatic Diboronic Acids	Meropenem, imipenem, ceftazidime	CMY-2, CMY-2, KPC-3	8–16 fold	KP, EC, PA	[75]
QPX7728	Tebipenem, ceftibuten, amdinocillin	OXA-48, CMY-2	4–32 fold	*Enterobacterales*	[76]
BLI-489	Imipenem, meropenem	KPC-2, KPC-2, OXA-23	16 fold	*Enterobacterales*	[77]
D63, D2148, D2573	Imipenem, meropenem	KPC-2, CTX-M-15, SHV-1, TEM-1, NDM-1, Amp-C	16 fold	EC	[78]
2-Mercaptomethyl thiazolidines	Imipenem	SHI-1	4 fold	EC	[79]

EC: *Escherichia coli*; KP: *Klebsiella pneumoniae*; PA: *Pseudomonas aeruginosa*; MIC: minimum inhibitory concentration; MIC: minimum inhibitory concentration.

**Table 8:** Analysis of IC_50_ values of serine beta-lactamases inhibitors.

Compounds	Substrates	Enzymes	Activity IC_50_	Pathogens	Ref.
A-amido-b-triazolylethaneboronic acid transition state	Not specified	KPC2, CTX-M-96, CTX-M-15	2–135 nM	EC	[80]
VNRX-7145	Ceftibuten	SHV-5, KPC-2, AmpC, OXA-48	0.003–8.55 μM	EC, KP	[81]
3-aryl substituted benzoxaborole derivatives	Meropenem	KPC-2, AmpC, TEM-1	86 nM	EC, KP	[82]
Aulfahydantoin derivatives	Not specified	TEM-1, TEM-15	130–510 μM	not specified	[83]

EC: *Escherichia coli*; KP: *Klebsiella pneumoniae*; MIC: minimum inhibitory concentration; IC50: half maximal inhibitory concentration.

**Table 9:** Classification and overview of β-lactamase inhibitors.

Category	Representative Compounds	Mechanism of Action	Characteristics	Clinical Application
Serine β-lactamase Inhibitors (SBLIs)	Clavulanic acid	Forms covalent bonds with the active site of serine β-lactamases, irreversibly inhibiting enzyme activity.	Highly effective and broad-spectrum, effective against various serine β-lactamases.	Commonly used in combination with penicillins or cephalosporins, such as amoxicillin-clavulanate.
	Sulbactam	Same as above.	Stable and effective against various serine β-lactamases.	Often combined with ampicillin, such as ampicillin–sulbactam.
	Tazobactam	Same as above.	Highly effective against various serine β-lactamases.	Frequently paired with piperacillin, such as piperacillin–tazobactam.
	Avibactam	Reversibly inhibits enzyme activity through non-covalent interactions.	Broad-spectrum, effective against various serine β-lactamases.	Often used with ceftazidime, such as ceftazidime–avibactam.
	Vaborbactam	Reversibly inhibits enzyme activity through non-covalent interactions.	Broad-spectrum, effective against various serine β-lactamases.	Commonly used with meropenem.
Metallo-β-lactamase Inhibitors (MBLIs)	Taniborbactam	Chelates zinc ions in the enzyme’s active site, inhibiting enzyme activity.	Broad-spectrum, effective against various metallo-β-lactamases.	Often used with cefepime, effective against NDM, VIM, etc.
	Xeruborbactam	Chelates zinc ions, inhibiting enzyme activity.	Broad-spectrum, effective against various metallo-β-lactamases.	Commonly used with cefepime, effective against NDM, VIM, etc.
	RPX7009	Chelates zinc ions, inhibiting enzyme activity.	Broad-spectrum, effective against various metallo-β-lactamases.	Often used with meropenem.
Novel Inhibitors	Piperacillin/Tazobactam/Avibactam	Combines multiple inhibitory mechanisms to enhance activity against multidrug-resistant strains.	Multiple inhibitory mechanisms, highly effective against multidrug-resistant bacteria.	Used for treating infections caused by multidrug-resistant bacteria.
	Other novel compounds (e.g., certain peptide inhibitors)	Inhibits β-lactamase activity through various mechanisms.	Under development, with potential high efficacy and low toxicity.	In clinical trial stages, with future prospects for treating resistant bacterial infections.

## Data Availability

Not applicable.
